# Using an analytic rubric system for the evaluation of anterior ceramic crown preparation performed by preclinical dental students

**DOI:** 10.1371/journal.pone.0318752

**Published:** 2025-04-03

**Authors:** Mohammed M. Al Moaleem, Hafiz A. Adawi, Nasser M. Alahmari, Mohammed E. Sayed, Nassreen H. Albar, Abdulmajeed Okshah, Abdullah A. Meshni, Thrya S. Gadah, Abdulkarim H. Alshehri, Bandar M.A. AL-Makramani

**Affiliations:** 1 Department of Prosthetic Dental Science, College of Dentistry, Jazan University, Jazan, Saudi Arabia; 2 Department of Prosthodontics, College of Dentistry, King Khalid University, Abha, Saudi Arabia; 3 Restorative Dental Science Department, College of dentistry, Jazan University, Jazan, Saudi Arabia; 4 Department of Allied Dental Health Sciences, College of Applied Medical Sciences in Khamis Mushait, King Khalid University, Abha, Saudi Arabia; Danube Private University, AUSTRIA

## Abstract

**Background:**

This study aimed to assess the performance of preclinical dental male and female students in performing maxillary anterior all-ceramic preparations using a validated analytic rubric system.

**Methods:**

An analytical rubric for the assessment of maxillary anterior ceramic preparations was used in a preclinical phase study with two evaluators. The grading was based on a 4-point scale for all steps for crown preparation, and the total score was calculated out of 40 points. Differences in rubric parameter scores between male and female students were analyzed via an independent t test. Other tests, such as the kappa test and Pearson’s correlation coefficient, were used to measure the associations among grade point average (GPA), evaluators, and gender participants, with p <  0.050 considered a cutoff point for statistical significance.

**Results:**

The study revealed that students of both genders demonstrated high performance, with females scoring a mean of 37.70 ± 1.61 (SD) and males scoring a mean of 36.50 ± 2.36 (SD). A significant difference in the average overall score was recorded by both evaluators (p =  0.034). The highest value was for intact gingival margins and surrounding tissues (3.89 ± 0.31 males, 3.92 ± 0.28 females), and the lowest value was for axial wall reduction (3.50 ± 0.36 males, 3.62 ± 0.42 females). Among the different parameters, only the finish line form parameter was significantly different (p =  0.011), as found by evaluator 1. Inter-rater reliability analysis revealed varying levels of agreement between the evaluators. Strong positive correlations were found between several rubric parameters, but students’ GPA had a weak, nonsignificant correlation with their preclinical performance (p =  0.495).

**Conclusion:**

The analytic rubric revealed minor gender differences in preclinical performance, with female students performing slightly better. The rubric demonstrated varying inter-rater reliability and positive correlations across parameters. However, GPA showed limited correlation with preclinical performance.

## Introduction

Dental students must develop the knowledge, skills, and attitudes necessary to be competent and independent practitioners after their undergraduate years, as they will be responsible for providing invasive and irrevocable treatments to patients in the future [1]. The assessment of students’ knowledge and skills is important for educators, as it allows them to evaluate the effectiveness of their teaching methods in producing competent dental graduates. Before entering clinical sessions, dental students undergo preclinical sessions to practice on models, and the assessment of their preclinical performance is essential for future patient safety and to provide feedback on teaching methods [2].

The assessment of student performance has traditionally followed a visual system, with individual grades or quantitative division into smaller scores [3]. Typically, preclinical courses require assessments by different faculty examiners, which aim to objectively reflect both students’ knowledge and performance [4]. For consistency and standardization among examiners to be maintained, the assessment procedure should provide validity, reliability, effectiveness, and efficiency. Furthermore, the purpose of the assessment should be clear to both the assessor and the assessed student [5]. The evaluation should also provide immediate and comprehensive feedback to students, enabling them to learn from their performance [6].

The American Dental Education Association recommends the use of multiple assessment methods to evaluate preclinical skills and competence effectively, with the ultimate goal of developing the skills needed for effective clinical and preclinical practice [7]. The assessment approaches used in preclinical prosthodontic training vary widely, with the most prevalent methods being the subjective “glance and grading” approach and more structured checklists based on objective criteria [8]. Both approaches are attractive for higher education and are still commonly used to evaluate dental students’ practical skills, although this subjective approach has been criticized because it lacks objectivity and does not effectively promote student learning [9]. The literature indicates that poorly designed rating scales without clear, objective criteria can lead to significant inter-rater variability [9]. Rater bias and incorrect interpretation of the rating scale are also major contributors to the marked variance in these assessment methods [10].

Learning is facilitated when students receive feedback from assessment methods that are consistent and based on meaningful, explicit criteria [11,12]. A lack of uniformity, uncertainty regarding the importance of assessment, and a lack of well-defined assessment criteria are some of the more commonly expressed concerns among students [13]. Rubrics create clear guidelines for assessment and define the criteria for performance [11].

Many studies have demonstrated the positive impact that well-designed rubrics have on dental students’ learning [14,15]. O’Donnell et al. proposed that one way to objectify the assessment process could be through the use of rubrics which is a scaled tool with levels of achievement and clearly defined criteria [11]. Rubrics can improve the objectivity and consistency of evaluations across different faculty members, provide students with detailed constructive feedback on their performance and empower them to self-assess their own work against the established criteria [11]. Research has shown that the use of rubrics in dental education can lead to higher levels of inter-rater reliability compared to more subjective evaluation methods [14].

In dentistry, information regarding the use of rubrics in the evaluation of students in preclinical, clinical, and presentation tasks in different specialties, classes, and preclinical and clinical phases is scarce. Although no studies have used rubrics to evaluate preclinical anterior all-ceramic crown preparation, previous studies have used them for oral presentation assessment in orthodontics [16], periodontics [17], evaluation of endodontic procedures [14,15,18] and students’ self-assessment performance in preclinical endodontics [19] and conservative dentistry courses [20].

The fixed prosthodontic course at the Faculty of Dentistry, Jazan University, was introduced to fourth-year undergraduate students. As part of this course, students must complete a series of preclinical tasks before being allowed to deal with patients. During their preclinical years, students perform ceramic crown preparation procedures on ivory teeth attached to artificial jaws according to meticulously designed rubrics for each step.

A well-constructed rubric is a permanent reference for clinical performance guidelines, while some studies have delved into the impact of rubrics on student performance, the research in this area is still in its nascent stage. Dental students must acquire the skills, knowledge, and attitudes necessary for independent practice. Preclinical courses are integral to this process, ensuring competency in clinical procedures before managing real patients. Reliable assessment methods are essential to evaluate the effectiveness of teaching strategies and ensure patient safety.

This study aimed to evaluate the scores achieved by fourth-year preclinical dental students (both male and female) on an all-ceramic preparation for a maxillary right central incisor (tooth #12), completed as part of the final practical exam during the 2023–2024 academic year. By applying a validated and enhanced analytic rubric system, the study also aimed to address gender-based differences in performance. Additionally, correlations between the general point average (GPA) and both evaluators according to gender were assessed. The null hypothesis stated that there would be no significant differences in the analytic rubric scores between male and female fourth-year dental students at the College of Dentistry, Jazan University, Saudi Arabia, regarding the steps and performance involved in the preclinical preparation of an all-ceramic maxillary anterior central incisor.

## Participants and methods

### Study design and ethical approval

This cross-sectional, double-blinded study was conducted at the Department of Prosthetic Dental Sciences, College of Dentistry, Jazan University, for preclinical-phase dental students (fourth year) in both male and female sections. The study was approved by the ethical committee of the College of Dentistry, Jazan University under # CODJU-2321F on August 21, 2023, and by the Standing Committee for Scientific Research - Jazan University (Approval No. REC-45/03/761) on September 22, 2023. This study was conducted from 25 September to 03 December 2023. The present study adhered to the STrengthening the Reporting of OBservational studies in Epidemiology (STROBE) guidelines.

### Participants

Students who registered in this preclinical course in the academic year of 2023–2024 were included in this analytic rubric evaluation study. Students were informed of the study’s objectives and agreed to use their evaluation for the prepared all-ceramic crown preparations for maxillary anterior typodont teeth in this study. An informed consent was obtained from all students to participate in this study. Students were informed that their participation is voluntary and they can withdraw at any time without any impact on their academic standing. Two evaluators with 12 years of prosthodontics teaching experience assessed the preparations after calibration sessions to ensure consistency. Both evaluators have extensive expertise in teaching practical skills, particularly those related to crown preparation.

### Study and investigation type

In the present study, the identities of the participating students were kept confidential from the examiners, and the examiners’ identities were not released to the contributing students. This approach ensured that bias would not impact the outcome of the study, as some of the examiners were involved in teaching the same students during the whole course. The contributing fourth-year preclinical phase students prepared a full all-ceramic retainer preparation on the maxillary right central incisor for their end-semester exams as a requirement of the course. The contributing male and female students were given clear guidelines regarding the exercise type and were informed about the analytic rubric criteria for the assessment; they also exercised in the same manner earlier during the same academic year.

### The inclusion and exclusion criteria

The study included 53 fourth-year dental students (28 males and 25 females) enrolled in the preclinical fixed prosthodontics course. Participants who completed prerequisite courses, all course requirements and consented to their data being used were included. Students who failed to complete the preparation within 90 minutes or modified their work outside the phantom head setup were excluded. After the end of the exercise, the analytic rubric evaluation sheets were collected and numbered before scoring by the two evaluators began.

### Teeth mounting and preparation

The ivory teeth were mounted in the typodont model (Frasaco, Tettnang, Germany), and all-ceramic full retainers were carried out on tooth #12. All the models were mounted on the phantom heads during all steps of preparation [21]. According to these preclinical course requirements, students should finish preparing and restoring different types of full and partial retainers for both maxillary and mandibular teeth and anterior and posterior teeth. All the prepared ivory teeth were numbered according to the students’ serial numbers.

### Data sources and rubric parameter scoring

This analytic rubric for the assessment of preclinical maxillary anterior all-ceramic preparation was used for the assessments of students. An analytic rubric is a structured assessment tool that breaks down performance into multiple detailed criteria, with each scored independently. This differs from general rubrics, which provide a single overall score without assessing individual components. The rubric criteria were developed through a review of existing rubrics reported in the literature and iterative discussions with prosthodontic faculty members. The criteria aimed to comprehensively assess key aspects of ceramic crown preparation. The rubric was pilot-tested with faculty members not directly involved in the study, and their feedback, along with that of students, was incorporated into the final version. This process helped refine the criteria and enhance clarity. Prior to the commencement of the assessments, the examiners underwent a calibration session to ensure consistency in applying the rubric criteria. This session involved reviewing and discussing sample student preparations to align their assessments with the rubric standards. Both evaluators independently scored the same set of prepared samples, and their scores were compared to assess inter-rater agreement. Based on the results, minor adjustments were made to ensure alignment. This calibration session was not repeated during the study, as it was deemed sufficient to ensure reliable evaluations for the current cohort. They have significant experience in teaching prosthodontics, particularly in developing practical and clinical skills.

The evaluators independently assessed the students’ all-ceramic retainer preparation details directly after each step of the preclinical examination. No time limit was specified for the completion of the scoring. However, examiners were requested to perform the assessments alone and not in groups. The analytic rubric used in the study was based on a 4-point scale for the assessment of the maxillary right central incisor. The scoring of each parameter was based on the following parameters: (1) incisal reduction, (2) Height-Width Ratio, (3) two-plane reduction, (4) axial wall reduction on the four surfaces: buccal, palatal, mesial, distal (B, P, M, D), (5) finish line form (B, P, M, D), (6) finish line location (B, P, M, D), (7) finish line continuity (B, P, M, D), (8) total occlusal convergence (TOC), (9) intact gingival margins and surrounding tissues, (10) preservation and protection of adjacent teeth [Table 1]. The grading was calculated as excellent, good, average, or unsatisfactory and scored as 4, 3, 2, or 1; then, the sum was calculated out of the total (40 points) for each student.

**Table 1:** Rubric parameters used in preclinical evaluation of maxillary anterior ceramic crown preparation.

Parameter	Level of achievement	Grades	Obtained Score
**Incisal Reduction**	Optimal reduction of 2.0 mm	Excellent (4)	
	Moderately under-reduced < 2 mm or moderately over-reduced < 2mm	Good (3)	
	Severely under-reduced < 1.5 mm or severely over-reduced < 2.5 mm	Average (2)	
	Inappropriate incisal reduction and takes help by the instructor	Unsatisfactory (1)	
**Height-Width Ratio**	Perfectly preparation with 0.4	Excellent (4)	
	Height-Width Ratio is slightly affected	Good (3)	
	Height-Width Ratio is severely affected	Average (2)	
	Does not adhere to height and width ratio at all	Unsatisfactory (1)	
**Two plane reduction**	Proper two-plane reduction present; Providing adequate material bulk for strength/ aesthetic	Excellent (4)	
	Proper two-plane reduction but slightly improper; Over or under-reduced	Good (3)	
	Proper two-plane reduction but significantly improper; Over or under-reduced	Average (2)	
	Fails to prepare two plane and takes help from instructor	Unsatisfactory (1)	
**Axial walls reduction (B,P,M,D)**	Optimal reduction of 1–1.5 mm on all four axial walls and rounded line and points angle	Excellent (4)	
	Moderately over-reduced or under-reduced axial walls, lack of rounded line or points angle	Good (3)	
	Severely over-reduced or under-reduced axial walls, lack of rounded line or points angle	Average (2)	
	Fails to reduce the axial walls and takes help from instructors	Unsatisfactory (1)	
**Finish line form** **(B,P,M,D)**	Perfectly prepares the finish line in all four surfaces (1 mm facial and 1mm on palatal)	Excellent (4)	
	Moderately prepares the finish line on buccal and palatal surfaces	Good (3)	
	Severely over or under reduced finish line in all of the four surfaces	Average (2)	
	Fails to prepare any finish line and takes helps from instructor	Unsatisfactory (1)	
**Finish line location** **(B,P,M,D)**	Optimal margins placement 0.5 mm supra-gingivally	Excellent (4)	
	Overextended ˃0.5 mm subgingival or moderately underextended ˃1mm subgingival	Good (3)	
	Overextended ˃1.0 mm subgingival or underextended ˃1.5 mm supragingival	Average (2)	
	Fails to prepare the finish line and takes helps from instructor	Unsatisfactory (1)	
**Finish line continuity** **(B,P,M,D)**	Optimal: Margins are smooth, continuous, and well defined	Excellent (4)	
	Moderate roughness of margins, slight non-continuous & moderate lack of definitions	Good (3)	
	Significantly roughness of margins, non-continuous & lack of definitions of finish line	Average (2)	
	Fails to prepare the finish line continuity and takes helps from instructor	Unsatisfactory (1)	
**Total occlusal convergence** **(TOC)**	Optimal taper: Retentive walls have 6^0^ of taper with no undercuts between axial walls	Excellent (4)	
	Moderate over-taper of 10^0^–20 ^0 ^or moderate undercuts between axial walls	Good (3)	
	Severe over-taper of ˃20 ^0 ^or with sever undercuts visually present in two axial walls	Average (2)	
	Severe under or over-taper with sever undercuts present in four axial walls	Unsatisfactory (1)	
**Intact gingival margins and surrounding tissues**	Perfectly breaks the contact with gap of 0.6mm in both mesial and distal sides	Excellent (4)	
	Breaks the contact with gap of 0.6mm in either mesial or distal sides	Good (3)	
	Contact is broken with a gap of 0.2–0.4 mm in either mesial or distal sides	Average (2)	
	Contact is not broken in both mesial and distal sides	Unsatisfactory (1)	
**Preservation and protection of adjacent teeth**	Adjacent teeth in both mesial and distal side are unaffected	Excellent (4)	
	Adjacent teeth in either mesial or distal side are moderately damaged	Good (3)	
	Adjacent teeth in both mesial and distal side are severely damaged	Average (2)	
	Adjacent teeth in both mesial and distal side are severely damaged	Unsatisfactory (1)	
**Total grade gained**			**Out of/40**

This analytic rubric resembled a grid in which the parameters are listed in the leftmost column and with levels of scoring (performance) listed across the row by using numbers along with descriptive tags. Each parameter was scored individually, and the rightmost column was filled with the particular score for each preparation. The sum of all the scores for all the parameters was then taken as the total score of each male and female student (Table 1). Individual printed log-book sheets were used for each student’s assessment, and the hard copies were numbered according to the student university identification card assigned to the dentoforms for all the contributing students.

### Grade point average for participants

A copy of the contributors of the preclinical phase students’ total GPA marks was collected and obtained from their Registration Unit at the college. Table 2 shows the criteria reference scale used by the College of Dentistry, Jazan University [22]. The data were categorized in relation to males and females and analyzed.

**Table 2:** Criteria reference for general point average for clinical performance.

Grade range	GPA (out of 5)	Values	Values in symbols
95–100	5.0	Exceptional	A^+^
From 90 to <95	4.75	Superior	A
From 85 to <90	4.50	Excellent	B^+^
From 80 to <85	4.0	Very good	B
From 75 to <80	3.5	Above average	C^+^
From 70 to <75	3	Good	C
From 65 to <70	2.5	Pass- high	D^+^
From 60 to <65	2.0	Pass	D
<60	1.0	Faill	F

### Inter- evaluators agreement and Intra- evaluator’s reliability

For consistency between and within evaluators to be ensured, inter-evaluator agreement between the two evaluators and intra-evaluator reliability for the parameters were assessed. Kappa tests and reliability tests were used to calculate kappa and inter-item correlation coefficient (ICC) values [23]. The results showed that the content reliability of the overall scale was internally consistent. The value of the reliability coefficient of the data collection tool (rubric) was between 85% and 87%, indicating that the items (criteria, level of achievements, and rating) used in the rubric are internally consistent.

### Data processing and analysis

The Statistical Package for the Social Sciences (SPSS) version 26.0 (IBM Corp., Armonk, NY, USA) was used for statistical analysis. The mean and standard deviation (±SD) were calculated to describe the rubric parameters of preclinical prosthodontics. Independent t tests were used to test for significant differences in the scores of the rubric parameters of preclinical fixed prosthodontics between the male and female groups. Kappa tests and reliability tests were used to assess inter-evaluator agreement and intra-evaluator reliability. Finally, Pearson’s correlation coefficient test was used to measure the associations among GPA, evaluators and participants. Here, p <  0.050 was considered the cutoff point for statistical significance.

## Results

A total of 53 students (28 males and 25 females) participated in this preclinical fixed prosthodontics course, which was conducted with fourth-year dental students. The rubric parameters of preclinical fixed prosthodontics were used and evaluated by two evaluators. The female students scored slightly higher than the male students did on most parameters, but the differences were not statistically significant (p >  0.050), only the finish line form parameter (evaluated by evaluator 1) showed a statistically significant difference between female and male students (p =  0.011) [Table 3]. The total scores out of 40 for the first and second evaluators were slightly higher for females than for males and reported as means with standard deviations (SD): 37.92 ± 2.29 (females, evaluator 1), 37.48 ± 1.81 (females, evaluator 2), 36.46 ± 3.20 (males, evaluator 1), and 36.54 ± 2.87 (males, evaluator 2), respectively.

**Table 3:** Mean (±SD) for each rubric parameter of preclinical fixed prosthodontics by evaluator and gender.

Parameter	Evaluator 1			Evaluator 2		
	Male	Female	P value^a^	Male	Female	*p*-value^a^
Incisal Reduction	3.64 (±0.49)	3.68 (±0.48)	0.780	3.61 (±0.57)	3.64 (±0.57)	0.834
Height-Width Ratio	3.57 (±0.57)	3.68 (±0.48)	0.455	3.46 (±0.58)	3.60 (±0.50)	0.363
Two plane reduction	3.61 (±0.57)	3.68 (±0.48)	0.613	3.79 (±0.42)	3.88 (±0.33)	0.365
Axial walls reduction (B,P,M,D)	3.46 (±0.58)	3.68 (±0.56)	0.172	3.54 (±0.51)	3.56 (±0.51)	0.863
Finish line form (B,P,M,D)	3.57 (±0.50)	3.88 (±0.33)	0.011	3.57 (±0.57)	3.80 (±0.41)	0.098
Finish line location (B,P,M,D)	3.79 (±0.42)	3.88 (±0.33)	0.365	3.89 (±0.31)	3.92 (±0.28)	0.740
Finish line continuity (B,P,M,D)	3.57 (±0.57)	3.80 (±0.41)	0.098	3.61 (±0.57)	3.72 (±0.54)	0.462
Total occlusal convergence (TOC)	3.54 (±0.58)	3.72 (±0.54)	0.236	3.57 (±0.57)	3.76 (±0.44)	0.181
Intact gingival margins and surrounding tissues	3.93 (±0.26)	4.00 (±0.00)	0.161	3.86 (±0.36)	3.84 (±0.37)	0.865
Preservation and protection of adjacent teeth	3.79 (±0.42)	3.92 (±0.28)	0.170	3.64 (±0.49)	3.76 (±0.44)	0.360
**Total scores**	36.46 (±3.20)	37.92 (±2.29)	0.061	36.54 (±2.87)	37.48 (±1.81)	0.154

^a^Independent T Test Buccal (B), Palatal (P), Mesial (M), Distal (D).

The overall analytic rubric score for female students was slightly higher than that for male students for most parameters, but the differences were not statistically significant (p >  0.050), except for the finish line form parameter, which was evaluated by both evaluators and was significantly different (p =  0.011) [Table 4].

**Table 4:** Overall mean and SD for each rubric parameter of preclinical fixed prosthodontics for both evaluators by gender.

Parameter	Overall or TOTAL Evaluator 1 and 2		
	Male	Female	*p*-value^a^
Incisal Reduction	3.63 (±0.38)	3.66 (±0.47)	0.769
Height-Width Ratio	3.52 (±0.42)	3.64 (±0.37)	0.264
Two plane reduction	3.70 (±0.44)	3.78 (±0.33)	0.431
Axial walls reduction (B,P,M,D)	3.50 (±0.36)	3.62 (±0.42)	0.269
Finish line form (B,P,M,D)	3.57 (±0.42)	3.84 (±0.31)	0.011
Finish line location (B,P,M,D)	3.84 (±0.24)	3.90 (±0.20)	0.322
Finish line continuity (B,P,M,D)	3.59 (±0.39)	3.76 (±0.36)	0.101
Total occlusal convergence (TOC)	3.55 (±0.48)	3.74 (±0.44)	0.144
Intact gingival margins and surrounding tissues	3.89 (±0.21)	3.92 (±0.19)	0.620
Preservation and protection of adjacent teeth	3.71 (±0.25)	3.84 (±0.24)	0.068
**Overall average scores**	36.50 (±2.36)	37.70 (±1.61)	0.034

^a^Independent T Test.

In terms of the overall average analytic rubric score, female students scored 37.70 ± 1.61 higher than male students did 36.50 ± 2.36 according to both evaluators, with a statistically significant difference in the overall average analytic rubric score (p =  0.034) [Table 5].

**Table 5:** Gender differences in student groups by the different evaluators for the rubric parameters.

Evaluator	Gender	Mean ± SD	*p*-value^a^
**Total Evaluator 1**	Male	36.46 (±3.20)	0.061
	Female	37.92 (±2.29)	
**Total Evaluator 2**	Male	36.54 (±2.87)	0.154
	Female	37.48 (±1.81)	
**Overall average**	Male	36.50 (±2.36)	0.034
	Female	37.70 (±1.61)	

^a^Independent T Test.

Table 6 shows the kappa test results for the preclinical fixed prosthodontics analytic rubric parameters, which indicate varying levels of agreement between the two evaluators. Moderate agreement was observed for incisal reduction (0.305), two-plane reduction (0.335), and total occlusal convergence (0.343). The finish line form had fair agreement (0.280); however, several parameters showed no agreement, including the Height-Width Ratio (−0.016), axial wall reduction (0.033), finish line location (−0.138), finish line continuity (−0.122), intact gingival margins (−0.064), and preservation of adjacent teeth (−0.252).

**Table 6:** Kappa test for analytic rubric parameters of preclinical fixed prosthodontics.

Parameter		Evaluator 2										
		Incisal Reduction	Height-Width Ratio	Two plane reduction	Axial walls reduction	Finish line form	Finish line location	Finish line continuity		Total occlusal convergence (TOC)	Intact gingival margins and surrounding tissues	Preservation and protection of adjacent teeth
**Evaluator 1**	**Incisal Reduction**	0.305	0.078	0.186	0.066	0.020	−0.071		−0.092	0.075	0.028	0.135
	**Height-Width Ratio**	0.681	−0.016	0.493	−0.067	−0.118	−0.073		−0.226	0.182	0.253	−0.091
	**Two plane reduction**	0.636	−0.021	0.335	−0.109	−0.180	−0.168		−0.206	0.128	0.276	−0.067
	**Axial walls reduction**	0.348	0.081	0.138	0.033	−0.030	−0.170		−0.173	0.255	0.260	−0.083
	**Finish line form**	0.059	0.183	0.048	0.331	0.280	−0.165		0.200	0.338	0.188	0.134
	**Finish line location**	−0.084	0.161	−0.071	0.155	0.189	−0.138		0.045	0.412	0.090	−0.073
	**Finish line continuity**	0.083	0.009	−0.064	−0.001	−0.004	−0.162		−0.122	0.100	0.172	0.028
	**Total occlusal convergence**	0.199	0.032	0.105	0.060	−0.042	−0.162		−0.154	0.343	0.233	−0.264
	**Intact gingival margins and surrounding tissues**	0.145	−0.073	0.128	−0.075	−0.070	−0.057		−0.067	−0.070	−0.064	0.047
	**Preservation and protection of adjacent teeth**	0.042	−0.124	−0.050	−0.131	−0.087	−0.131		−0.233	0.151	−0.178	−0.252

Strong positive correlations were observed between the rubric scores of axial wall reduction and total occlusal convergence (TOC) (0.783), incisal reduction and two-plane reduction (0.647), the Height-Width Ratio and two-plane reduction (0.632), the Height-Width Ratio and intact gingival margins and surrounding tissues (0.522), axial wall reduction and finish line form (0.551), finish line form and finish line location (0.575), and finish line continuity and preservation and protection of adjacent teeth (0.664) [Table 7]. Moreover, a few parameters or rubric scores presented moderate positive correlations, such as the incisal reduction and Height-Width Ratio (0.453), axial wall reduction and finish line continuity (0.424), finish line form and finish line continuity (0.444). The remaining parameter rubric scores show either low and weak positive correlations or negative correlations.

**Table 7:** Inter-Item correlation coefficient for Rubric parameters.

Parameter	Incisal Reduction	Height-Width Ratio	Two plane reduction	Axial walls reduction	Finish line form	Finish line location	Finish line continuity	Total occlusal convergence (TOC)	Intact gingival margins and surrounding tissues	Preservation and protection of adjacent teeth
**Incisal Reduction**	1.000	0.453	0.647	0.392	0.146	−0.156	−0.063	0.314	0.222	0.082
**Height-Width Ratio**		1.000	0.632	0.377	0.331	0.115	0.041	0.359	0.522	−0.066
**Two plane reduction**			1.000	0.229	0.097	−0.134	−0.049	0.319	0.359	−0.034
**Axial walls reduction**				1.000	0.551	0.367	0.424	0.783	0.259	0.331
**Finish line form**					1.000	0.575	0.444	0.368	0.059	0.362
**Finish line location**						1.000	0.100	0.324	−0.070	0.229
**Finish line continuity**							1.000	0.271	0.025	0.664
**Total occlusal convergence**								1.000	0.253	0.198
**Intact gingival margins and surrounding tissues**									1.000	−0.051
**Preservation and protection of adjacent teeth**										1.000

Strong and significant correlations were found between the GPA and total analytic rubric score of evaluator 1 and the overall average analytic rubric score of both evaluators (r =  0.826 and p <  0.001) and between the total analytic rubric score of evaluator 2 and the overall average analytic rubric score of both evaluators (r =  0.751 and p <  0.001) [Table 8]. The GPA has a weak and nonsignificant correlation with the total scores of evaluators 1 (r =  0.020, p =  0.886) and 2 (r =  0.141, p =  0.313). A weak and nonsignificant correlation with the overall average score (r =  0.096, p =  0.495) was observed. By contrast, the total score of the first evaluator has a strong and highly significant correlation with the overall average score (r =  0.826, p <  0.001). Similarly, the total score of the second evaluator also has a strong and highly significant correlation with the overall average score (r =  0.751, p <  0.001) (Table 8).

**Table 8:** Correlations between GPA, evaluators and participants.

		GPA	Total evaluator1	Total evaluator2	Overall average
**GPA**	Pearson Correlation	1	0.020	0.141	0.096
	Sig. (2-tailed)		0.886	0.313	0.495
**Total evaluator1**	Pearson Correlation		1	0.248	0.826**
	Sig. (2-tailed)			0.073	< 0.001
**Total evaluator2**	Pearson Correlation			1	0.751**
	Sig. (2-tailed)				< 0.001
**Overall average**	Pearson Correlation				1
	Sig. (2-tailed)				

**. Correlation is significant at the 0.01 level (2-tailed).

## Discussion

In preclinical educational settings, the assessment provided by faculty members serves as the standard that students use to evaluate their own performance. One key challenge is that students may not be able to reliably conduct qualitative self-assessments to the same level of accuracy as experienced faculty members. Variations in interfaculty grading can hinder students from developing a consistent self-assessment standard, but this situation can be improved via appropriate faculty calibration [24]. Over the last few years, a number of articles have suggested that rubric-based assessment can enhance instructional quality, improve grading consistency, and support student achievement [11,12,14,15,25]. Thus, this study investigated the use of an analytical rubric-based assessment scheme for evaluating the performance of all-ceramic crown preparations for maxillary right central incisors in fourth-year preclinical dental students.

The current study revealed that female students scored an overall average analytic rubric score of 37.70 ± 1.61 (mean ±  SD), which was higher than male students’ score of 36.50 ± 2.36 (mean ±  SD), as assessed by both evaluators. This difference in the overall average analytic rubric score was statistically significant, with a p-value of 0.034. While similar gender differences in performance have been reported in other studies, such as those examining academic or practical skill outcomes, we acknowledge that our study specifically measures students’ practical skills in crown preparation, not self-efficacy as reported by Brookhart et al. [26]. The null hypothesis is partially accepted since there was no gender significant differences between the two evaluators separately, but the overall average between gender was significant in the rubric scores for all ceramic preparation foe maxillary central incisors, during preclinical setting.

A study conducted by Kornmehl et al. in the U.S. revealed that male students significantly overestimate themselves compared with their female counterparts, leading to significantly greater differences in their grading scores between males and females [27]. Also, similar findings recorded recently [28]. However, our results differ from the findings reported by Mobaraki et al., who reported that male students presented a greater percentage of excellent performance in various parameters, such as incisal reduction, axial wall preparations, and planar reduction, with significant differences observed between genders [21]. Similarly, Moaleem et al. reported significant differences in the axial taper reduction created by male and female students [29]. However, direct comparisons between the current study and these previous investigations are limited, as standardized rubrics in the assessment were not used during their evaluation process.

In addition, Habib 2018 investigated the use of an analytic rubric system for the evaluation of all-ceramic crown preparations. This study examined various parameters, including time management, axial reduction, occlusal reduction, two-plane reduction, finish of margins and walls, and preservation of adjacent teeth. The results revealed significant differences in examiner scores across most of these parameters, except for time management. The two-plane reduction parameter showed the greatest variability in scoring among examiners. In contrast, the finishing of the preparation margins and walls resulted in the least difference in the assessments. Moreover, the performance of students in axial reduction and preservation of adjacent teeth were identified as the weakest areas during crown preparations [30]. These findings were consistent with the results of the present study.

According to the rubric, the examiners demonstrated fair to moderate agreement in the grading of some parameters. However, for the Height-Width Ratio, axial wall reduction, finish line location, finish line continuity, intact gingival margins and surrounding tissues, and preservation and protection of adjacent teeth parameters, no agreement was established, with kappa values ranging from − 0.252 to − 0.016, similar to the results of other studies [30]. These parameters appear to be the most challenging for examiners to assess visually, as the estimation of these parameters is fairly difficult and prone to both underestimation and overestimation [31]. Visual inspection may not be consistent, and faculty members sometimes mark unacceptable student work as acceptable, leading to grading inconsistencies [32]. Additionally, inter-rater agreement can be impacted by the complexity of the rubric criteria, variation in subject responses, and the range of scores used with more scoring criteria, response variability, and wider score ranges, leading to less rater alignment and consistency [33].

For these issues to be addressed, digitally assisted evaluation via 3D inspection and metrology software has been considered an alternative to conventional visual inspection. Additionally, software-based automated evaluation (SAE) could eliminate subjective rater biases and provide more reliable and precise assessments. Studies have shown that evaluation consistency can be improved via SAE, as it minimizes human-based errors in measurement [24]. Han et al. demonstrated that evaluation via a software-based automated protocol demonstrated perfect intra-rater agreement, and evaluation via a digitally assisted evaluation protocol revealed moderate-to-good intra-rater agreement [34]. Although these digital methods provide students with consistent, accurate, and objective assessments, they are costly and time consuming to design, and they require training to provide reliable results. These newer and more sophisticated methods for evaluating student performance still require precise tooth preparation measurements to function properly and provide meaningful evaluations of student work [32].

The retention and strength of cement-retained crowns depend on various factors, such as the convergence angle of the preparation, the height of the preparation, and the preparation surface. An optimal convergence angle of 6° was recommended. When the convergence angle is less than 6°, undercutting might lead to insertion problems. Conversely, a convergence greater than 16° compromises retention [35]. The lowest scoring criteria were axial wall reduction (avg. 3.50 ± 0.36 for males, 3.62 ± 0.42 for females) and total convergence angle (avg. 3.55 for males, 3.74 for females), which are considered clinically acceptable; other studies support our findings [21,29]. Interestingly, both of these parameters achieved the highest correlation (r = 0.783) compared with the correlations of the other parameters. Proper axial reduction is essential for good functional morphology and structural durability. Such reductions are important, as the optimum bulk of material must be present in the restoration to prevent any fracture or perforation after cementation [36]. Vertical depth grooves are created on the buccal surface via a tapered diamond bur, followed by removal of the tooth structure between the grooves. The depth grooves help control the amount of reduction. Rosella et al. reported that controlling the depth and direction of tooth tissue removal is the most challenging aspect for prosthodontists [37].

Academic performance was determined to be a strong predictor of health promotion skills, as students with a high GPA in their theoretical coursework were 3.4 times more likely to exhibit competence in this domain than those with an average GPA [38].

The present study revealed a weak and nonsignificant correlation between students’ GPA and the total scores of evaluators 1 and 2 and the overall average score (p =  0.886, p =  0.313 and p = 0.495, respectively). This result was in accordance with previous research, such as the study by Bindayel, which reported no significant correlation between instructors’ grading and students’ final course grades [16].

In general, students appreciate clear guidelines to help them complete performance-based assessments successfully [39]. In the present study, the assessment form provided students with specific criteria across five categories, which were introduced and supplied to them prior to their preparations. The detailed rubric was intended to serve both as a guide for instructors’ assessments and as a tool for student learning during their preparatory process.

The primary goal of preclinical dental education is to prepare students to deliver the highest quality of care to patients in the clinical setting. Students build upon the foundation established in preclinical courses, and this learning carries forward into their advanced clinical training. Research has shown that preclinical performance positively correlates with clinical success [25]. However, managing a preclinical prosthodontic course presents challenges, particularly in providing timely and personalized feedback to students due to the limited time available during and outside of scheduled lab sessions. While dental students have competing academic responsibilities, which can complicate the scheduling of meetings, this is primarily an organizational challenge that can be addressed through effective planning and scheduling [11]. Using analytic rubrics for tooth preparation assessment can provide students with instant and objective feedback, which allows them to identify weaknesses and follow up with faculty at their convenience to address those areas [12].

Further research is needed to better understand the relationships between gender, GPA, and the use of rubrics as assessment tools on the quality of anterior all-ceramic restorations performed by students. Additionally, comparative analyses are recommended to evaluate the effectiveness of analytical rubric parameters compared with other evaluation methods, such as digitally assisted evaluation, checklists [40], software-based automated evaluation [34], global rating scales [10], structured rating scales [41], and the impact of enhanced personal protective equipment on the experience of the student operator and the restorative procedure [42]. Furthermore, strong recommendation for using a computer-assessed evaluation methods such as prep-check supported self-assessment [43,44], and self-assessment method [6,20,27,28].

We correlated the students’ GPA with their practical performance to explore any potential relationship between academic performance and practical skills. However, we acknowledge that GPA primarily reflects cognitive abilities and may not be the most appropriate measure for assessing practical psychomotor skills. Therefore, we suggest that future studies should correlate practical exam performance with previous practical assessments in earlier dental courses or didactic exam results from the fixed prosthodontics subject to gain more relevant insights into the relationship between practical skills and academic performance.

The analytic rubric-based assessment criteria presented in this study are intended to provide a comprehensive framework for developing and implementing a preclinical dental skill assessment for anterior all-ceramic crown preparation. However, its applicability may be limited to similar technical dental procedures and may not readily extend to other types of clinical skill assessments. Importantly, this study has several limitations. First, the assessment criteria assume a certain level of expertise and resources on the part of the instructors and institutions implementing the assessment. Therefore, this approach may not be feasible for all institutions or instructors to follow the certain as outlined. Second, although our study offers valuable insights into developing and validating an assessment sheet for preclinical evaluation of anterior all-ceramic crown preparation, it was conducted by a single dental faculty member. As such, the influence of individual differences among instructors and students within our institution may affect our findings’ generalizability. Third, the current rubric’s detailed criteria provide comprehensive guidance but may be time-consuming for evaluators during busy sessions. A concise version of the rubric is recommended for future implementation to streamline the evaluation process. Additionally, this study found a statistically significant difference in the overall average analytic rubric score between male and female students (p =  0.034), with female students performing better. However, this result may be influenced by factors specific to this cohort, such as differences in teaching methods or assessment conditions. Future longitudinal studies with successive cohorts are recommended to determine whether this trend is consistent over time or unique to this group. Expanding the study to include other institutions could also enhance the generalizability of these findings.

## Conclusion

The study revealed that minor, albeit non-significant, gender differences were observed, with female students achieving slightly higher scores. The rubric showed varying levels of inter-rater reliability, with some parameters demonstrating moderate agreement while others presented lower consistency. Additionally, GPA showed a weak, non-significant correlation with practical performance, suggesting that theoretical knowledge may not directly predict hands-on skills in this context.

## Supporting information

S1 File Preclinical student evaluation data set.
(XLSX)

## References

[1] Holzinger A, Lettner S, Franz A. Attitudes of dental students towards patients with special healthcare needs: Can they be improved? Eur J Dent Educ. 2020;24(2):243–51. doi: 10.1111/eje.12490 31845452 PMC7328724

[2] McGleenon EL, Morison S. Preparing dental students for independent practice: a scoping review of methods and trends in undergraduate clinical skills teaching in the UK and Ireland. Br Dent J. 2021;230(1):39–45. doi: 10.1038/s41415-020-2505-7 33420457 PMC7791324

[3] Al Amri MD, Sherfudhin HR, Habib SR. Effects of evaluator’s fatigue and level of expertise on the global and analytical evaluation of preclinical tooth preparation. J Prosthodont. 2016;27(7):636–43. doi: 10.1111/jopr.12558 27809403

[4] Deeb JG, Koertge TE, Laskin DM, Carrico CK. Are There Differences in Technical Assessment Grades Between Adjunct and Full-Time Dental Faculty? A Pilot Study. J Dent Educ. 2019;83(4):451–6. doi: 10.21815/JDE.019.046 30745344

[5] Manogue M, Kelly M, Bartakova Masaryk S, Brown G, Catalanotto F, Choo-Soo T, et al. 2.1 Evolving methods of assessment. Eur J Dent Educ. 2002;6 Suppl 3:53–66. doi: 10.1034/j.1600-0579.6.s3.8.x 12390260

[6] Karaman P. Effects of using rubrics in self-assessment with instructor feedback on pre-service teachers’ academic performance, self-regulated learning and perceptions of self-assessment. Eur J Psychol Educ. 2024;39(3):2551–74. doi: 10.1007/s10212-024-00867-w

[7] Kramer GA, Albino JEN, Andrieu SC, Hendricson WD, Henson L, Horn BD, et al. Dental student assessment toolbox. J Dent Educ. 2009;73(1):12–35. doi: 10.1002/j.0022-0337.2009.73.1.tb04636.x 19126764

[8] Elmaroush M, Ben Hamida S, Elgarboae Z, Mohsen N. Variability in assessment of preclinical prosthodontics work of undergraduate student. Khalij-Libya J Dent Med Res. 2022;6(2):129–33. doi: 10.47705/kjdmr.2262007

[9] Ilgen JS, Ma IWY, Hatala R, Cook DA. A systematic review of validity evidence for checklists versus global rating scales in simulation-based assessment. Med Educ. 2015;49(2):161–73. doi: 10.1111/medu.12621 25626747

[10] Henrico K, Makkink AW. Use of global rating scales and checklists in clinical simulation-based assessments: a protocol for a scoping review. BMJ Open. 2023;13(5):e065981. doi: 10.1136/bmjopen-2022-065981 37173107 PMC10186456

[11] O’Donnell JA, Oakley M, Haney S, O’Neill PN, Taylor D. Rubrics 101: a primer for rubric development in dental education. J Dent Educ. 2011;75(9):1163–75. doi: 10.1002/j.0022-0337.2011.75.9.tb05160.x 21890846

[12] Jonsson A, Svingby G. The use of scoring rubrics: Reliability, validity and educational consequences. Educational Research Review. 2007;2(2):130–44. doi: 10.1016/j.edurev.2007.05.002

[13] Licari FW, Knight GW, Guenzel PJ. Designing evaluation forms to facilitate student learning. Journal of Dental Education. 2008;72(1):48–58. doi: 10.1002/j.0022-0337.2008.72.1.tb04452.x 18172235

[14] Escribano N, Belliard V, Baracco B, Da Silva D, Ceballos L, Fuentes MV. Rubric vs. numeric rating scale: agreement among evaluators on endodontic treatments performed by dental students. BMC Med Educ. 2023;23(1):197. doi: 10.1186/s12909-023-04187-3 36998034 PMC10061985

[15] Abiad RS. Rubrics for practical endodontics. J Orthod Endod. 2017;03(01). doi: 10.21767/2469-2980.100039

[16] Bindayel NA. Reliability of rubrics in the assessment of orthodontic oral presentation. Saudi Dent J. 2017;29(4):135–9. doi: 10.1016/j.sdentj.2017.07.001 29033521 PMC5634801

[17] Satheesh KM, Brockmann LB, Liu Y, Gadbury-Amyot CC. Use of an analytical grading rubric for self-assessment: a pilot study for a periodontal oral competency examination in predoctoral dental education. J Dent Educ. 2015;79(12):1429–36. doi: 10.1002/j.0022-0337.2015.79.12.tb06042.x 26632297

[18] Tenkumo T, Fuji T, Ikawa M, Shoji S, Sasazaki H, Iwamatsu-Kobayashi Y, et al. Introduction of integrated dental training jaw models and rubric criteria. Eur J Dent Educ. 2019;23(1):e17–31. doi: 10.1111/eje.12395 30306676

[19] Tsai S-T, Ho Y-C, Tsai C-L, Yang S-F, Lai Y-L, Lee S-Y. Evaluation of students’ self-assessment performance in preclinical endodontic training by means of rubrics and a 3D printed model. J Formos Med Assoc. 2022;121(11):2203–10. doi: 10.1016/j.jfma.2022.03.021 35484003

[20] Mittal P, Jadhav GR, Pawar M, Banerjee S, Wangaskar S, Di Blasio M, et al. Impact of self-assessment on dental student’s performance in pre-clinical conservative dentistry course. BMC Oral Health. 2024;24(1):593. doi: 10.1186/s12903-024-04140-w 38778282 PMC11112931

[21] M Mobarki Y, M Bajawi A, O Hakami A, A Mobaraky A, A Darraj O, M Halawi S, et al. Evaluation of undergraduate dental students in typodont preparation for all-ceramic crowns. J Int Med Dent. 2018;5(2):72–9. doi: 10.18320/jimd/201805.0272

[22] Ramos RB, Belza MSL, Coronado GB, Alwadai GS, Alamoudi NA, Abogazalah NN, et al. A Comparative analysis of introvert and extrovert dental students toward clinical performance. J Contemp Dent Pract. 2024;25(9):830–5. doi: 10.5005/jp-journals-10024-3668 39791409

[23] Koo TK, Li MY. A Guideline of selecting and reporting intraclass correlation coefficients for reliability research. J Chiropr Med. 2016;15(2):155–63. doi: 10.1016/j.jcm.2016.02.012 27330520 PMC4913118

[24] Miyazono S, Shinozaki Y, Sato H, Isshi K, Yamashita J. Use of digital technology to improve objective and reliable assessment in dental student simulation laboratories. J Dent Educ. 2019;83(10):1224–32. doi: 10.21815/JDE.019.114 31182626

[25] Doğan CD, Uluman M. A comparison of rubrics and graded category rating scales with various methods regarding raters’ reliability. Educ Sci Theor Pract. 2017;17(2):631–51. doi: 10.12738/estp.2017.2.0321

[26] Brookhart SM, Chen F. The quality and effectiveness of descriptive rubrics. Edu Rev. 2014;67(3):343–68. doi: 10.1080/00131911.2014.929565

[27] Kornmehl DL, Patel E, Agrawal R, Harris JR, Ba AK, Ohyama H. The effect of gender on student self-assessment skills in operative preclinical dentistry. J Dent Educ. 2021;85(9):1511–7. doi: 10.1002/jdd.12638 33990132

[28] Liang L, Nagasawa M, Ha V, Lin AJ, Akiba Y, Akiba N, et al. Association between gender and self-assessment skills amongst Japanese dental students. J Dent Sci. 2024;19(3):1533–9. doi: 10.1016/j.jds.2023.12.025 39035302 PMC11259621

[29] Al-Moaleem M, Shariff M, Porwal A, AlMakhloti E, Tikare S. Evaluation of the degree of taper and convergence angle of full ceramo-metal crown preparations by different specialists centers at Assir Region, Saudi Arabia. Saudi J Med Med Sci. 2015;3(3):198. doi: 10.4103/1658-631x.161996

[30] Habib SR. Rubric system for evaluation of crown preparation performed by dental students. Eur J Dent Educ. 2018;22(3):e506–13. doi: 10.1111/eje.12333 29498157

[31] Mays KA, Crisp HA, Vos P. Utilizing CAD/CAM to Measure Total Occlusal Convergence of Preclinical Dental Students’ Crown Preparations. J Dent Educ. 2016;80(1):100–7. doi: 10.1002/j.0022-0337.2016.80.1.tb06063.x 26729690

[32] Schepke U, van Wulfften Palthe ME, Meisberger EW, Kerdijk W, Cune MS, Blok B. Digital assessment of a retentive full crown preparation-An evaluation of prepCheck in an undergraduate pre-clinical teaching environment. Eur J Dent Educ. 2020;24(3):407–24. doi: 10.1111/eje.12516 32072741 PMC7508182

[33] AlHumaid J, Tantawi ME, Al-Ansari AA, Al-Harbi FA. Agreement in Scoring Preclinical Dental Procedures: Impact on Grades and Instructor-Related Determinants. J Dent Educ. 2016;80(5):553–62. doi: 10.1002/j.0022-0337.2016.80.5.tb06115.x 27139206

[34] Han S, Yi Y, Revilla-León M, Yilmaz B, Yoon H-I. Feasibility of software-based assessment for automated evaluation of tooth preparation for dental crown by using a computational geometric algorithm. Sci Rep. 2023;13(1):11847. doi: 10.1038/s41598-023-39089-3 37481612 PMC10363138

[35] Amine M, Wahid HO, Fahi S, Lehmouddi S, Hamza M, Elarabi S. Assessment of Convergence Angle of Tooth Preparations for Complete Crowns Among Dental Students: Typodont vs Simulator. Int J Dent. 2022;2022:7615892. doi: 10.1155/2022/7615892 35592253 PMC9113901

[36] Al-Moaleem MM, Al Hashim NS, Asiri KA, Al Makhloti EA, Al Ahmari NM, Tikare S. Assessment of porcelain fused to metal crown preparations by general practitioners in Saudi Arabia. J Adv Med Med Res. 2015;7(2):116–123. doi: 10.9734/BJMMR/2015/15861

[37] Rosella D, Rosella G, Brauner E, Papi P, Piccoli L, Pompa G. A tooth preparation technique in fixed prosthodontics for students and neophyte dentists. Ann Stomatol (Roma). 2016;6(3–4):104–9. doi: 10.11138/ads/2015.6.3.104 26941898 PMC4755680

[38] Gudipaneni RK, Ganji KK, Almaeen SH. Competency assessment in pediatric dental practice: A study on recent dental graduates in integrated curriculum. J Dent Educ. 2024;88(12):1720–9. doi: 10.1002/jdd.13677 39075766

[39] Olson J, Krysiak R. Rubrics as tools for effective assessment of student learning and program quality. In book: Curriculum Development and Online Instruction for the 21st Century. 2021. 173–200. ISBN 978-1-7998-7655-7. doi: 10.4018/978-1-7998-7653-3.ch010

[40] McKinley RK, Strand J, Ward L, Gray T, Alun-Jones T, Miller H. Checklists for assessment and certification of clinical procedural skills omit essential competencies: a systematic review. Med Educ. 2008;42(4):338–49. doi: 10.1111/j.1365-2923.2007.02970.x 18338987

[41] Uoshima K, Akiba N, Nagasawa M. Technical skill training and assessment in dental education. Jpn Dent Sci Rev. 2021;57:160–3. doi: 10.1016/j.jdsr.2021.08.004 34567290 PMC8449262

[42] Miletic V, Avuthu R, Zaprzala P, Divnic-Resnik T, Savic-Stankovic T, Cabunac J, et al. Enhanced personal protective equipment and dental students’ experience and quality of a restorative procedure in a simulated clinical setting. J Dent Educ. 2024;88(11):1490–502. doi: 10.1002/jdd.13593 38795331

[43] Wolgin M, Grabowski S, Elhadad S, Frank W, Kielbassa AM. Comparison of a prepCheck-supported self-assessment concept with conventional faculty supervision in a pre-clinical simulation environment. Eur J Dent Educ. 2018;22(3):e522–9. doi: 10.1111/eje.12337 29575669

[44] Wolgin M, Frank W, Kielbassa AM. Development of an analytical prepCheck-supported approach to evaluate tutor-based assessments of dental students’ practical skills. Int J Comput Dent. 2018;21(4):313–22. 30539173

